# The role of blood purification therapies in the treatment of chronic kidney disease–associated pruritus: a systematic review

**DOI:** 10.1093/ckj/sfae266

**Published:** 2024-08-29

**Authors:** Matteo Marcello, Davide Marturano, Claudio Ronco, Monica Zanella

**Affiliations:** Department of Nephrology, Dialysis and Trasplantation, San Bortolo Hospital, Vicenza, Italy; International Renal Research Institute Vicenza, IRRIV, Vicenza, Italy; Department of Nephrology, Dialysis and Trasplantation, San Bortolo Hospital, Vicenza, Italy; International Renal Research Institute Vicenza, IRRIV, Vicenza, Italy; International Renal Research Institute Vicenza, IRRIV, Vicenza, Italy; Department of Nephrology, Dialysis and Trasplantation, San Bortolo Hospital, Vicenza, Italy; International Renal Research Institute Vicenza, IRRIV, Vicenza, Italy

**Keywords:** chronic kidney disease-associated pruritus, dialysis, extracorporeal blood purification, hemadsorption

## Abstract

Chronic kidney disease–associated pruritus (CKD-aP) is a common complication in dialysis patients which is not fully addressed by pharmacological and dialytic therapy. The objective was to review the literature on the effects of extracorporeal blood purification modalities on CKD-aP. The population comprised patients aged ≥18 years on chronic dialysis. PubMed, Embase, and Medline were systematically searched until February 2024 for clinical studies comparing the effect of different dialysis modalities on pruritus intensity. Two reviewers extracted data independently. Risk of bias for randomized controlled trials (RCTs) was assessed using the Cochrane tool. Any extracorporeal blood purification therapy for the treatment of CKD-aP was included. Outcome was quantitative change in pruritus intensity on a validated itching scale. This review included eight RCTs examining five different dialysis modalities, three observational studies examining three dialysis modalities, and six prospective clinical trials assessing four dialysis modalities. These treatments included peritoneal dialysis, low-flux and high-flux dialysis, hemodiafiltration, expanded hemodialysis, hemadsorption, hemodiafiltration with endogenous reinfusion and dialysis with polymethylmethacrylate membrane. Risk of bias was high in most studies. The largest body of evidence was found for the efficacy of hemadsorption. Limitations of evidence included heterogeneity in diagnostic tools and treatment, risk of selection bias, small sample sizes and short follow-up durations that made it challenging to perform a robust systematic review and meta-analysis. Despite the high prevalence of pruritus among dialysis patients, current evidence for efficacy of standard dialytic treatment is weak. The only technique that appears to be effective is hemoadsorption alone or coupled with hemodialysis. More high-quality studies are needed to confirm the long-term benefits.

## INTRODUCTION

Chronic kidney disease–associated pruritus (CKD-aP), previously referred to as uremic pruritus, is a common and debilitating complication of CKD that negatively impacts patients’ quality of life causing sleep disturbance, depression and social isolation [1, 2] and it is associated with higher mortality [3].

**Figure 1:**
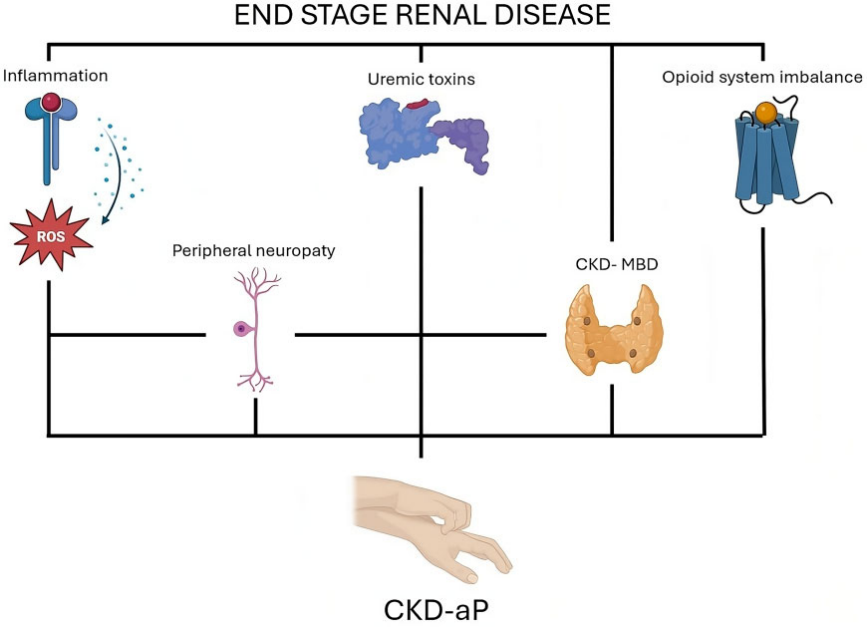
Pathogenesis of CKD-aP. Elevated levels of inflammatory cytokines, such as interleukin-2, interleukin-6 and tumor necrosis factor-alpha, have been observed in patients with CKD-aP. These cytokines can sensitize peripheral nerves and stimulate the release of pruritogenic mediators. Protein-bound uremic toxins (PBUT) such as p-cresol sulfate and indoxyl sulfate have a relevant role in CKD-aP. These molecules can induce PAR-2 expression in the cultures of human epidermal keratinocytes that have been associated with the intensity of pruritus assessed by VAS score. The impairment of small myelinated and unmyelinated sensory nerves observed in CKD may result in hyperexcitability and abnormal transmission of itch signals to the central nervous system. The imbalance in the stimulation and antagonism of mu and kappa opioid receptors may contribute to the dysregulation of itch perception and transmission. CKD–Mineral Bone Disorder (CKD-MBD) has been historically associated with pruritus in dialysis patients, although its role in the pathogenesis of CKD-aP is controversial.

The pathogenesis is not fully understood. As shown in Fig. 1, the current understanding is a multifactorial etiology [4] that includes the accumulation of uremic toxins [5], inflammation [6], altered sensory nerve function [7] and opioid system imbalance [8, 9]. Other risk factors are reported to contribute to the development of CKD-aP, although this is not supported by a strong level of evidence like CKD–mineral bone disorder [10], elevated serum magnesium [11] and aluminum concentrations [12], and ultrafiltration volume [13].

Usually, the first line of treatment for itching in dialysis patients involves conservative measures such as adequate hydration, use of emollients and treatment of underlying conditions [14]. However, these approaches often provide only partial relief, and more targeted therapies such as anti-histamines, capsaicin and μ-opioid receptor antagonists are needed for those refractory to the above therapies [15, 16].

The prevalence of moderate to severe itching among dialysis patients only decreased from 46% in 1996 to 37% in 2015 [17]. However, the true prevalence may be underestimated due to the lack of standardized diagnostic criteria and the subjective nature of the symptom. This is responsible for undertreatment—data from an international registry indicates that 18% of patients with pruritus received no treatment [18].

Blood purification techniques have been explored as potential therapies for managing itching. However, CKD-aP still has a high prevalence in dialysis patients that has only partially been influenced by the achievement of adequate dialysis and more biocompatible membranes [19]. While these modalities may offer some benefits, their precise role in the comprehensive treatment remains an active area of investigation [20].

This systematic review aims to synthesize the evidence on the role of blood purification therapies in the treatment of CKD-aP in dialysis patients and assess the strength of data to provide a clinical guide for dialytic management of this condition.

## MATERIALS AND METHODS

### Data sources, search strategy, eligibility criteria

To perform our narrative systematic review, we searched on MEDLINE (articles from 1946), EMBASE (from 1974) and PubMed (from 1965) databases, our selection timeline goes until 3 February 2024. Two reviewers independently performed study selection, data extraction and bias analysis.

The search strategy utilized keywords “hemodialysis,” “haemodialysis” or “dialysis” unified by the AND operator term to “itching,” “itch*” or “prurit*,” to include extracorporeal treatment and not exclude peritoneal dialysis (PD). The search strategy was the same for all databases selected and is available in Supplement 1. Citations were collected and managed with Zotero software.

We included observational and experimental trials, retrospective and prospective, randomized and not, that enrolled adult patients (age ≥18 years) on chronic dialysis. Studies included patients with pruritus-related to end-stage kidney disease (ESKD), while those with pruritus from other causes were excluded. Our selection excluded trials with drugs, lotions and behavior interventions. Furthermore, we only accepted articles assessing pruritus with validated scales (Table 1) [21–27] or validated questionnaires on CKD symptoms [28, 29]. Papers without details on kidney replacement treatment were deleted. We also excluded papers published in languages other than English.

**Table 1:** Validated itching scores [21–27].

Itching scale	Description
VAS score	The VAS consisted of a horizontal line with “no pruritus (0 mm)” at the left end and “worst possible pruritus (100 mm)” at the right end. The distance from the left end to the vertical line (mm) was determined.
Numerical Rating Scale (NRS)	Scale from 1 to 10 to evaluate the patient-report intensity of worst itch in the previous 24 h. Mild (0–3); moderate (4–6); severe (7–10)
Brief Itching Inventory	Patients filled 1 of 11 bubbles on a human figure diagram [“0 (itch does not interfere); 1, 2, 3, 4, 5, 6, 7, 8, 9 and 10 (itch completely interferes)”] for each of the questions. The total score is the sum of the numeric value of each answered question
Skindex-10	A shorter version of the validated Skindex-16, which recalls symptoms from the previous week; the total score is the sums of disease, mood/emotional distress and social functioning domains. Each question is graded in intensity from 0 to 6
5D itch scale	The scale assess duration, degree, direction, distribution and disability associated with itching in the prior 2 weeks. Total score ranges between a minimum of 5 points (no itching) and maximum of 25 points (maximum severity). This scale correlates strongly with the VAS score and is able to detect significant changes in pruritus over the 6-week follow-up period
Shiratori Severity Score	The scale assess separately for daytime and nighttime pruritus. Daytime is evaluated on the following 5-point scale: 0 = “no itching at all” (no symptoms), 1 = “tolerable without scratching” (very mild), 2 = “subsides after light scratching” (mild), 3 = “subsides after considerable scratching” (moderate) and 4 = “does not subside, prompting repeated scratching” (severe). Night-time pruritus is evaluated on the following 5-point scale: 0 = “no itching at all” (no symptoms), 1 = “slight itching at bedtime (very mild), 2 = “some itching, but subsides after scratching; don't wake up due to itchiness” (mild), 3 = “I wake up due to itching” (moderate) and 4 = “I can hardly sleep due to itching” (severe)
Duo's modified Detailed Pruritus Score (DPS)	The score (0–40) assess separately for morning and afternoon itching, according to extent of scratching, distribution range, frequency of attacks and sleep disturbance

### Data extraction, assessment risk of bias

**Figure 2:**
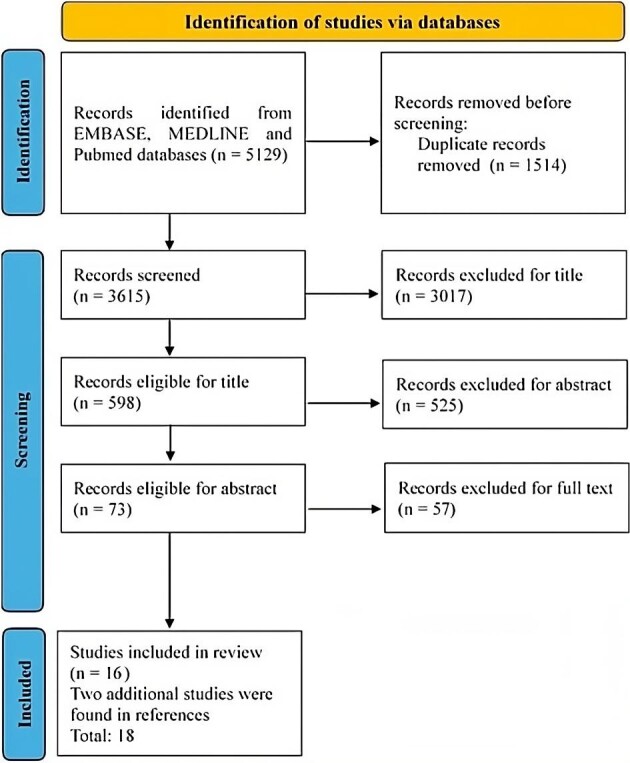
Search strategy.

Selection criteria are shown in Fig. 2. A total of 5129 studies were identified at first. Then we deleted duplicate papers coming from different databases, obtaining 3615 articles. We performed title screening, excluding 3017 articles and abstract screening deleting 525 articles. Of the remaining 73 studies, 56 did not meet our criteria; therefore, the final qualitative synthesis included 16 eligible studies (Table 2). In addition, we cited two studies found in the reference lists of our pool of articles that matched the criteria but were not reported in the search databases.

**Table 2:** Studies summary.

Study authors	Year	Type	Patients (n)	Itching scale	Treatment	HD membranes	Duration
Masi CM, Cohen EP	1992	Observational, cross-sectional	115	VAS	HD vs PD	CA; PS	T0
Aucella F, Vigilante M *et al*.	2007	Prospective	8	VAS	HD vs HFD	CA; PS; PAES; PMMA	8 months
Lin HH, Liu YL *et al*.	2008	Prospective	24	VAS	HD vs HFD	PS; CA; PMMA	4 weeks
Chen ZJ, Cao G *et al*.	2009	Prospective, randomized, double-blind	116	VAS	HD vs HFD	PS	12 weeks
Yong DSP, Kwok AOL *et al*.	2009	Prospective, cross-sectional	179	NRS	HD vs PD vs no RRT	Not specified	16 months
Ko MJ, Wu HY *et al*.	2013	Prospective	111	VAS	HD vs HFD	PS	53 months
Jin DH, Shen HY *et al*.	2014	Observational, prospective	67	Duo's modified scale	HD vs HD + HDF vs PD	CA; PS	12 weeks
Karkar A, Abdelrahman M, Locatelli F	2015	Prospective, randomized	72	KDQOL-SF	HFD vs HDF-post	PS; PAES	24 months
Wu HY, Peng YS *et al*.	2016	Observational, cross-sectional	380	VAS	HD vs PD	PS (HF or LF)	T0
Zhang J, Yuan Y *et al*.	2016	Prospective, randomized	40	VAS	HFD + HP vs HDF + HP	PAES; PS; resin	12 weeks
Li WH, Yin YM *et al*.	2017	Prospective, randomized	90	VAS, Duo's modified scale	HD vs HD + HP	PS; resin	8 weeks
Gu YH, Yang XH *et al*.^a^	2019	Prospective, randomized	158	NRS	HD vs HD + HP	Not specified; resin	2 years
Lim JH, Park Y *et al*.	2020	Prospective, randomized	49	KDQOL-SF	HFD vs HDx	PEAS-MCO; PS	12 weeks
Lengton R, van der Willik EM *et al*.	2022	Prospective, cohort	1926	KDQOL-36	HD vs PD	Not specified	Max 10 years
de Rooij ENM, Meuleman Y *et al*.	2022	Observational, prospective	456	DSI + Likert scale	ESKD vs HD/PD	Not specified	12 months
Zhao D, Wang Y *et al*.	2022	Prospective, randomized	438	Duo's modified scale	HD vs HFD vs HD + HP vs HFD + HP	PS, resin	12 months
Takahashi N, Mano J *et al*.^a^	2023	Prospective	20	VAS; Shiratori severity score	HDF	CA; PMMA	12 weeks
Zha F, Li W *et al*.	2023	Prospective, randomized	60	VAS; 5-D itch scale; 12-PSS	HFD vs HFD + HFR	PES; resin	24 weeks

^a^These studies were not present in databases screened but were found in studies references.

CA, cellulose acetate; PS, polysulfone; T0, time of observation; PAES, polyarylethersulfone; HF, high-flux; LF, low-flux; NRS, Numerical Rating Scale; RRT, renal replacement therapy; KDQOL-SF, Kidney Disease Quality of Life-Short Form; HP, hemoperfusion; DSI, dialysis symptoms index; 12-PSS, 12-Iter Pruritus Severity Scale; PES, polyethersulfone.

Risk of bias for randomized controlled trials (RCTs) was assessed by two authors independently, using the Cochrane Collaboration's tool [30] (Table 3). Disputes were resolved by consensus.

**Table 3:** Cochrane Collaboration's assessment of risk of bias tool.

Studies	Randomization process	Timing of recriutment	Blinding of patients and personnel	Blinding of outcome	Missing outcome data	Measurement of the outcome	Selection of the reported results	Overall risk
Chen ZJ, Cao G *et al*. 2009	U	L	L	L	L	L	L	U
Karkar A, Locatelli F 2015	U	L	H	L	L	H	H	H
Zhang J, Yuan Y *et al*. 2016	H	U	H	L	L	U	H	H
Li WH, Yin YM *et al*. 2017	H	L	H	L	L	H	L	H
Gu YH, Yang XH *et al*. 2019	H	L	L	L	L	U	L	H
Lim JH, Park Y *et al*. 2020	L	L	L	L	L	L	L	L
Zhao D, Wang Y *et al*. 2022	L	L	L	L	L	L	L	L
Zha F, Li W *et al*. 2023	L	L	L	L	L	U	L	U

H: High; L: Low; U: Unclear.

## RESULTS

### Dialysis initiation

Few studies have investigated the impact of dialysis initiation on the course of CKD-aP [31]. The European Quality (EQUAL) study is an ongoing, prospective, multicenter study that assessed the symptom burden in 456 ESKD patients ≥65 years using the dialysis symptom index. Patients have been studied during the year preceding and the year after dialysis initiation. At dialysis initiation, CKD-aP was one of the most commonly reported symptoms, with self-reported moderate-to-severe pruritus in about 30% of participants. After dialysis initiation, while the mean number of symptoms and burden decreased, the prevalence of itching did not improve. The results align with prior retrospective analyses [28, 32]; however, they come from a partial analysis of a non-randomized clinical trial.

### Dialysis adequacy

Given the high prevalence of persistent itching in chronic dialysis patients, research focused on interventions to improve dialysis adequacy as a potential strategy to reduce pruritus.

Ko *et al*. [33] conducted a 5 years prospective cohort study on 111 maintenance hemodialysis (HD) patients to assess the intensity of pruritus. Participants received 3.5–5.0 h of HD three times a week using bicarbonate dialysate. Dialysis adequacy was evaluated by Kt/V and normalized protein catabolic rate. At the end of follow-up, 111 patients remained and pruritus intensity was reported as aggravated in 17 patients (15.3%), unchanged in 37 patients (33.3%) and improved in 57 patients (51.4%). In multivariate analysis, lower Kt/V was the strongest predictor of moderate-to-severe pruritus, and a baseline Kt/V <1.5 was associated with aggravation of pruritus intensity. This longitudinal study has a follow-up long enough to exclude time-related bias, however patients were not randomized and adequacy parameters were assessed using a single-compartment model.

A recent prospective multicenter cohort study investigated the role of residual kidney function, dialysis adequacy and other biomarkers on pruritus in incidental dialysis patients (1256 in HD and 670 in PD) followed for a maximum of 10 years or until death or transplantation. Residual kidney function was expressed as estimated glomerular filtration rate, and dialysis adequacy was expressed as total Kt/V urea per week. The prevalence of pruritus was 70%, with 20% of patients describing severe symptoms. Higher residual kidney function and total weekly Kt/V at 12 months were found to be associated with lower burden score in both groups but more in PD patients. In contrast, no correlation was found between dialysis Kt/V dose and pruritus [34]. The study was not randomized and controlled, however it included a large cohort of patients allowing authors to compare HD with PD.

### PD vs HD

A lower prevalence of CKD-aP in PD patients compared with those on HD would be expected due to its continuous nature that may provide more efficient removal of uremic toxins and its higher preservation of residual kidney function. However, contrasting results have been reported in the literature with some studies showing no significant differences compared with HD [35] while other studies found lower prevalence among PD patients.

Wu *et al*. [36] conducted a cross-sectional study on 380 dialysis patients (84 PD patients and 296 HD patients) to assess the prevalence and characteristics of itching among these two groups. They did not find significant differences in the prevalence of pruritus (28.6% in PD patients vs 38.2% in HD patients), although the visual analog scale (VAS) score was significantly lower in the PD group (*P *= .04). However, this study carries some confounding factors. Patients on PD were fewer (22.1% vs 77.9%) and younger, with lower dialysis vintage and lower comorbidities. Moreover, a higher percentage of them achieved the target dose for dialysis adequacy. Furthermore, data regarding pruritus prevalence before dialysis initiation were not collected.

A prospective study evaluating 99 patients found that the severity of itching was significantly lower in the PD group compared with the HD group, with 18% vs 44% experiencing moderate-to-severe pruritus, respectively [37]. To further complicate the picture, data from RENINE/PROMs registry on 2978 dialysis patients showed that itching was present in approximately half of the patients and was more common in individuals receiving PD (59.4%) compared with HD (48.7%) [28].

Therefore, there are not sufficient data to unequivocally conclude that PD is superior to HD in the prevention or treatment of CKD-aP. Furthermore, dialysis adequacy metrics can vary greatly between PD and HD and this may partially explain the mixed results in comparing these two dialysis modalities.

### Impact of convective dialysis therapy on CKD-aP

Originally, membranes for HD were designed to remove small solutes such as urea and creatinine while avoiding albumin loss. These low-flux membranes provided effective clearance of small solutes through diffusion, but negligible clearance of middle molecules. Identifying these toxins and comprehending their role in developing several complications of CKD spurred new interest in developing synthetic high-flux and high-permeable membranes and their use in alternative techniques such as hemofiltration or hemodiafiltration.

**Figure 3:**
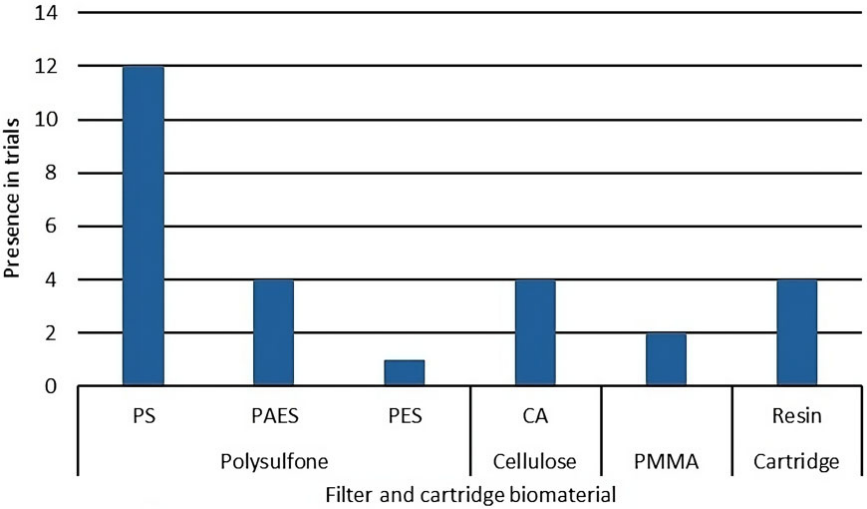
Material distribution among studies. PS, polysulfone; PAES, polyarylethersulfone; PES, polyethersulfone; CA, cellulose acetate.

Furthermore, poor membrane biocompatibility has been associated with increased inflammation and oxidative stress leading to the release of pruritogenic factors [38]. In the past, cuprophan membranes were used, but they were highly immunoreactive because of the large number of hydroxyl groups, responsible for complement activation and leukopenia. Technological advances made it possible to create synthetic membranes with reduced immunoreactivity. The graphic in Fig. 3 shows the frequency of the reported biomaterials of the extracorporeal blood circuits used in the studies included in this review.

#### High-flux HD

High-flux membranes composed of various membrane materials have been developed to remove small and middle solutes efficiently and have been used for convective therapy since the 1970s. We found heterogeneity in the definition of high-flux dialyzer in the various studies. However, according to the literature we considered membranes to be high-flux dialyzers when characterized by an Ultrafiltration Coefficient (Kf) of 20–40 mL/h/mmHg that allows a clearance of β2-microglobulin at a rate of >20 mL/min [39]. While several studies demonstrated higher removal of middle molecules and better clinical outcomes compared with low-flux dialyzers, there are few studies specifically examining their superiority in the management of CKD-aP.

In 2008, a randomized, prospective, double-blind study was performed to compare low-flux with high-flux dialysis. In this study by Chen *et al*. [40], 116 chronic dialysis patients were assigned to receive either conventional HD with low-flux membranes or high-flux HD over 6 months. The authors reported a significant decrease in pruritus intensity in the high-flux group compared with the low-flux group (*P *= .009) without significant differences in Kt/V values. Moreover, the high-flux HD (HFD) group had lower blood levels of β2-microglobulin (*P *= .012) and parathyroid hormone (PTH) (*P *= .01). This study demonstrated the superiority of high-flux dialyzers in controlling pruritus even in the presence of similar Kt/V values. The results are relevant because the study's population exceeded the number of patients needed according to 80% power and the risk of selection bias was low due to the randomized double-blind setting of the study.

#### Hemodiafiltration

Hemodiafiltration (HDF) is an advanced technique that combines the principles of diffusion and convection through a high-flux membrane, allowing for the removal of a wider range of solutes, including middle molecules. During HDF, the total ultrafiltered volume is the sum of the net ultrafiltration plus the replacement fluid infused to enhance convection.

While several studies demonstrated the superiority of hemodiafiltration over conventional HD in controlling complications of ESKD such as inflammation, anemia, cardiovascular events and peripheral neuropathy [41, 42], few clinical studies focused on the impact of HDF in the management of pruritus.

Karkar *et al.* [43] in 2015 conducted a single-center prospective randomized trial to assess the self-report health-related satisfaction level of 72 low-flux dialysis patients at baseline and after a randomized switch to either high-flux or online-HDF. The level of patient satisfaction was significantly higher in HDF group compared with high-flux dialysis. These effects were associated with significantly fewer episodes of hypotension, cramps and itching (*P *< .0001). These results are limited by the small number of patients and the use of a self-administered questionnaire, even though a validated one was used. Nonetheless, this was a prospective study with a follow-up of 24 months that included a cross-sectional analysis of the previous treatment period with low-flux membrane.

A clinical study conducted in China evaluated the impact of three different dialysis modalities on 67 elderly incident dialysis patients with a follow-up of 12 weeks. Patients were assigned to either conventional HD, HDF + HD or PD. Patients on HDF + HD underwent HDF treatment once a week and HD treatment twice a week. Before the initial dialysis, no differences in Kt/V, β2-microglobulin, iPTH, and pruritus score were found. After 12 weeks, the pruritus score decreased significantly in all groups except for the HD group. Furthermore, the authors reported a greater reduction of intact PTH, β2-microglobulin levels (*P *< .005), and pruritus scores (*P *< .001) in HDF and PD groups compared with the conventional HD group [44]. Results from this study are limited by its small sample size, short follow-up period and selection bias due to lack of randomization.

However, this technique, requires a large volume of substitution fluid, strict water quality management and optimal vascular access.

#### Expanded HD

More recently, another dialysis technique called expanded HD (HDx) has been developed using a novel medium cut-off membrane (MCO). These membranes have an increased pore size and tight pore size distribution to guarantee a steep sieving curve compared with high-flux dialyzers. These characteristics allow for enhanced removal of larger toxins with molecular weight up to 45 kDa, with only marginal albumin loss [45]. Furthermore, in MCO membranes, a significant degree of convection is possible due to a reduction in the inner diameters of the fibers and an increase in their length that enhances the process of internal filtration and backfiltration.

To date, only a few studies have assessed the impact of HDx on quality of life and symptom burden, with inconsistent results [46, 47]. However, the role of this technique in the management of CKD-aP has not been extensively studied yet.

In a recent prospective, controlled, open-label, phase 4 trial, 49 maintenance HD patients previously treated with HFD with Fresenius FX CorDiax 80 or 60 were randomly divided at a 1:1 ratio into MCO and HFD group. Patients in the first group switched to Theranova 400 dialyzer and those in the high-flux group continued HFD. The MCO group presented a higher morning pruritus intensity at baseline. However, after being treated with MCO membrane for 12 weeks, these patients presented similar morning pruritus intensity, smaller pruritus distribution in the morning (*P *= .034) and less frequent sleep disturbance (*P *= .023) compared with HFD group [48]. This study was designed, although not adequately powered. More robust, larger and longer-term studies are needed.

### Impact of hemadsorption on CKD-aP

In the last decades, the role of adsorption techniques in the management of uremic toxins has gained significant interest. Hemadsorption is a technique in which a sorbent, such as charcoal or resin, is placed in direct contact with blood in an extracorporeal circuit. Nonspecific sorbents attract molecules by exploiting chemical and physical interactions including van der Waals interactions, electrostatic attraction, hydrogen bonding and hydrophobic interactions. Hemadsorption can be performed either as a standalone therapy or coupled with dialysis, to enhance the removal of the larger molecules.

#### Polymethylmethacrylate membrane

Polymethylmethacrylate (PMMA) is a symmetric, highly biocompatible, synthetic dialysis membrane created in 1977 that combines diffusive and adsorptive clearance mechanisms. PMMA membranes have been shown to inhibit the production of inflammatory cytokines and have an increased adsorbing capacity for high molecular weight proteins, cytokines and free light chains. A specific type of these membranes is the BG series characterized by a weak anionic charge that enhances adsorption capacity [49].

Several studies demonstrated the beneficial effects of PMMA membranes on clinical outcomes, including improvement in pruritus, in CKD patients undergoing dialysis. In 2007, Aucella *et al*. [50] reported a decrease in pruritus score after 6 months of switching from low-flux dialysis to PMMA membrane along with a significant decrease in β2-microglobulin serum levels. Authors reported a decrease in VAS score of 15% after 1 month, 30% after 2 months and 55% after 6 months. However, this single-center study involved only eight patients who switched from low-flux dialyzer to PMMA membranes without randomization. Therefore, these results could be just the effect of the switch to a more permeable dialyzer and do not demonstrate the superiority of this dialysis modality compared with conventional HD.

A single-center trial on 30 patients with severe uremic pruritus showed that changing the dialysis prescription from conventional HD to PMMA membrane resulted in a significant improvement in pruritus symptoms over a 4-week period (*P *< .001). Interestingly, authors reported relief in pruritus already after 1 week [51]. Only 24 patients completed the study, 5 of them (>20%) were previously treated with low-flux dialyzer. Given the low sample size and the lack of randomization, these results alone do not represent a strong recommendation supporting the use of PMMA to treat CKD-aP. One interesting result in this study is that patients continued to manifest benefits from the treatment with PMMA membrane up to 8 weeks after changing the prescription back to the previous one. However, patients were not blinded on which dialyzer was used and this could represent a potential bias.

The most recent study addressing the issue comes from the group of Takahashi *et al*. [52]. The authors investigated the impact of switching from pre- or post-dilution online hemodiafiltration with an asymmetric triacetate membrane to post-dilution hemodiafiltration with PMMA membrane in 20 patients with moderate to severe itching. The results demonstrated a significant reduction in the pruritus score already after 2 weeks of dialysis with PMMA dialyzer but also a reduction in the proportion of patients with mild to moderate daytime pruritus assessed by Shiratori Severity Scale after 12 weeks. This study has several limitations including low sample size and the lack of control group which does not allow us to conclude that switching to PMMA membrane was the effective factor in the improvement of pruritus. However, this study assesses the impact of PMMA dialyzers in patients already treated with hemodiafiltration using the same modality. As a consequence, no differences were found in β2-microglobulin, α1-microglobulin and C-reactive protein reduction, therefore suggesting that the reduction in pruritus score was an additional effect of the new membrane.

These results come from small single-center studies, nonetheless they spurred further investigation on the impact of hemadsorption in the management of CKD-aP.

#### Hemadsorption

Despite an early interest in hemadsorption during the last decades of the previous century, the use of hemoadsorption in the treatment of uremic syndrome was limited due to poor biocompatibility and overwhelming side effects. However, with the more recent development of more selective and better-tolerated sorbents, this approach has gained renewed interest. Polystyrene-divinylbenzene is a neural, meso/macroporous, non-selective polymer used as a sorbent for newly developed cartridges (e.g. Cytosorb, Jafron series).

Several studies have shown the benefit of hemoadsorption in the management of CKD-aP. An observational study compared low-flux HD (LFHD) alone with LFHD + HA130 and LFHD + HA330, using the cartridges once every 2 weeks, for 2 h and with a blood flow of 180–200 mL/min in the management of itching. After 8 weeks of treatment, VAS score and modified Duo's pruritus score decreased significantly compared with the group without adsorption [53]. This study is limited by the comparison of hemadsorption with low-flux dialysis with a blood flow of 180–200 mL/min. The authors do not report data regarding dialysis adequacy. However, given the randomization of 90 patients into three groups, the superiority of HA330 cartridge compared with HA130 in reducing itching score may suggests a potential role of hemadsorption in the treatment of CKD-aP.

In one of them, 40 HD patients with uremic pruritus were randomly assigned to either HD + hemadsorption or hemodiafiltration + hemadsorption. After 12 weeks, the pruritus VAS score decreased significantly in both groups, but to a greater extent in the HDF + HA group (*P *< .05) [54]. Despite the randomization, authors do not report data on the previous dialysis prescription making difficult to interpret the reduction in pruritus scores and exclude a potential role of hemodiafiltration.

Another study followed 78 HD + HA patients and 80 conventional dialysis patients to assess CKD-aP. This study found that HA reduced pruritus score (*P *< .001) and improved sleep quality over 2 years [55]. This randomized study has a long follow-up. One limitation is the lack of data on the dialysis prescription before randomization.

In a prospective randomized, controlled, multicentre study, 440 patients were randomized to LFHD, high-flux HD (HFHD), LFHD with hemadsorption (LFHD-HA), and HFHD with hemadsorption (HFHD-HA). After 1 year of treatment, patients who underwent once-a-week hemadsorption, regardless of the dialyzer used, had >50% reduction in the pruritus score [56]. The strength of this study relies on the exclusion of the role of convection on itching, demonstrating an effective and independent role of hemadsorption in the management of CKD-aP.

#### Hemodiafiltration with endogenous reinfusion

Hemodiafiltration with endogenous reinfusion (HFR) is an advanced dialysis technique that combines diffusion, convection and adsorption. The technique involves two filters and a cartridge. A high-flux polyethersulfone dialyzer with a pore size >55 000 Da allows the removal of plasma water by high convective flux. The fluid obtained passes through a hydrophobic styrene resin with a large surface area (about 700 m^2^/g) and small pores (between 20 and 50 Å) that adsorb middle and protein-bound uremic toxins contained in high concentration. This fluid deprived of toxins is then used as a reinfusion fluid in a low-flux polyethersulfone membrane where diffusion and net ultrafiltration occur.

Different studies demonstrate the efficacy of HFR in the removal of middle molecules, cytokines and protein-bound uremic toxins [57, 58].

In a recent prospective randomized study, Zha *et al*. [59] investigated the efficacy of supra-HFR in the treatment of CKD-aP. In this study, 60 HD patients were divided into two groups: the control group received HD with high-flux membrane, while the experimental group received HFR. After 24 weeks, pruritus was significantly lower in patients undergoing HFR (*P *< .001). In both groups, one-third of patients were previously treated with low-flux dialyzers, however the reduction of VAS score was significantly higher in the study group compared with the high-flux group. This study was well conducted, however despite the randomization, the low sample size and short follow-up do not allow the support of strong evidence for the role of HFR in the treatment of CKD-aP.

## DISCUSSION

From the literature reviewed, while there is emerging evidence that blood purification techniques may play a role in the management of CKD-associated pruritus, there is a general lack of high-quality evidence to definitively establish the effectiveness of these approaches. The studies reviewed were generally small and frequently had methodological limitations that constrained the ability to draw strong conclusions. Furthermore, the heterogeneity of the diagnostic tools, dialysis regimens, populations and outcome measures used across studies makes it challenging to perform a robust systematic review and meta-analysis.

Risk of bias was high in half of the RCTs, while two studies raised some concerns in the randomization, allocation concealment and measurement of outcome. Only two studies assessed had a low risk of bias. One of them regarded HDx compared with high-flux dialysis, however this study, although well-designed had a small sample size and short follow-up. The largest body of evidence was found for the efficacy of hemadsorption with one study on 440 patients with a follow-up of 1 year.

In conclusion, the lack of diagnostic and therapeutic standardization is the major limitation. Further well-designed, adequately powered randomized controlled trials are needed to clarify the role of blood purification approaches in this clinical context. Therefore, this review does not allow recommendations on the choice between different modalities that should still consider the patient's needs and characteristics (e.g. vascular access, anticoagulation), the availability of various treatment options and the physician's experience (Table 4) [4].

**Table 4:** Treatment comparison.

Treatment	Efficacy	Tolerance	PBUT remotion	MMs remotion	Anticoagulation	Cost
HD	Low	Good	Low	Low	Standard	Low
HDF	Moderate	Good	Moderate	High	Standard	Moderate
HDx	Moderate	Good	Moderate	High	Standard	Moderate
HD + PMMA	High	Good	High	High	Standard	Moderate
HD + HP	High	Good	High	High	High dose	High
HFR	High	Good	High	High	High dose	High

PBUT, protein-bound uremic toxin; MMs, middle molecules; HP, hemoperfusion.

## Data Availability

No new data were generated or analysed in support of this research.
